# Sparse Regression in Cancer Genomics: Comparing Variable Selection and Predictions in Real World Data

**DOI:** 10.1177/11769351211056298

**Published:** 2021-11-27

**Authors:** Robert J O’Shea, Sophia Tsoka, Gary JR Cook, Vicky Goh

**Affiliations:** 1 Department of Cancer Imaging, School of Biomedical Engineering and Imaging Sciences, King’s College London, London, UK; 2 Department of Informatics, School of Natural and Mathematical Sciences, King’s College London, London, UK; 3 King’s College London & Guy’s and St Thomas’ PET Centre, St Thomas’ Hospital, London, UK; 4 Department of Radiology, Guy’s and St Thomas’ NHS Foundation Trust, London, UK

**Keywords:** Artificial intelligence, gene regulatory networks, models, statistical, computational biology, genomics

## Abstract

**Background:**

Evaluation of gene interaction models in cancer genomics is challenging, as the true distribution is uncertain. Previous analyses have benchmarked models using synthetic data or databases of experimentally verified interactions – approaches which are susceptible to misrepresentation and incompleteness, respectively. The objectives of this analysis are to (1) provide a real-world data-driven approach for comparing performance of genomic model inference algorithms, (2) compare the performance of LASSO, elastic net, best-subset selection, $L_{0}L_{1}$ penalisation and $L_{0}L_{2}$ penalisation in real genomic data and (3) compare algorithmic preselection according to performance in our benchmark datasets to algorithmic selection by internal cross-validation.

**Methods:**

Five large (n4000) genomic datasets were extracted from Gene Expression Omnibus. ‘Gold-standard’ regression models were trained on subspaces of these datasets ( n4000 , p=500 ). Penalised regression models were trained on small samples from these subspaces ( n∈{25,75,150},p=500 ) and validated against the gold-standard models. Variable selection performance and out-of-sample prediction were assessed. Penalty ‘preselection’ according to test performance in the other 4 datasets was compared to selection internal cross-validation error minimisation.

**Results:**

$L_{1}L_{2}$ -penalisation achieved the highest cosine similarity between estimated coefficients and those of gold-standard models. $L_{0}L_{2}$ -penalised models explained the greatest proportion of variance in test responses, though performance was unreliable in low signal:noise conditions. $L_{0}L_{2}$ also attained the highest overall median variable selection F1 score. Penalty preselection significantly outperformed selection by internal cross-validation in each of 3 examined metrics.

**Conclusions:**

This analysis explores a novel approach for comparisons of model selection approaches in real genomic data from 5 cancers. Our benchmarking datasets have been made publicly available for use in future research. Our findings support the use of $L_{0}L_{2}$ penalisation for structural selection and $L_{1}L_{2}$ penalisation for coefficient recovery in genomic data. Evaluation of learning algorithms according to observed test performance in external genomic datasets yields valuable insights into actual test performance, providing a data-driven complement to internal cross-validation in genomic regression tasks.

## Author Summary

Regression models are frequently used in cancer genomics, where they provide insight into the interactions between genes. Sparse regression models were developed to allow modelling of a large set of variables with a small number of samples – a scenario encountered frequently in genomics. However, evaluation of genomic model structures remains challenging, due to uncertainty regarding the true system of interactions. Previous studies have compared methods with synthetic data, which may not reflect the challenges of real-world data. In this study, genomic datasets were identified which contained enough samples to provide reasonable estimates of the true structures – which were used as ‘gold-standards’. Sparse regression methods were tasked with estimating the true structure given a small proportion of the available samples, allowing for comparison against the gold standards.

Our results show that the interaction strengths estimated by the $L_{1}L_{2}$ penalisation method correspond best with the gold standard models. Other penalisation methods, including the $L_{0}L_{2}$ penalisation method, may be unreliable in noisy data. We demonstrate that modelling decision may be supported by our evaluation method, an approach which may complement cross-validation.

## Background

### Regression models in cancer genomics

High-dimensional regression problems are ubiquitous in modern oncological research, as datasets often contain fewer observations than variables.^1-7^ The tractability of penalised regression approaches in this setting has led to a large volume of research into their applications.^1,7-9^ Penalised regression offers robust predictions in high dimensional data and mechanistic insights through the estimated coefficient vector.^1,7^ $L_{0}$ and $L_{1}$ penalties perform variable selection inherently, by shrinking small dependencies to zero.^9-11^ However, it is difficult to test the assumptions which penalised approaches require for valid model selection in real world datasets.^12,13^ Furthermore, standard model selection approaches such as cross-validation and the Bayesian information criterion may be unreliable for model selection in the high-dimensional setting.^14,15^

### Penalised regression

The inverse covariance matrix, ${\left(X^{T}X\right)}^{-1}$ , is undefined if n<p , precluding the use of ordinary least squared regression.^13,16^ Penalised regression methods facilitate modelling in the high-dimensional setting through the addition of bias terms. $L_{0}$ , $L_{1}$ and $L_{2}$ penalised linear regression may be generally formulated such that:

(1) $$\begin{array}{c} {\hat{\beta }}^{L_{0},L_{1},L_{2}}:=arg\underset{\left(\beta_{0},\beta \right)\in {\mathbb{R}}^{p+1}}{min}\left\{\begin{array}{l} \frac{1}{2}{\left\|y-\beta_{0}-X\beta \right\|}_{2}^{2}+\lambda_{0}{\left\|\beta \right\|}_{0}\\ +\lambda_{1}{\left\|\beta \right\|}_{1}+\lambda_{2}{\left\|\beta \right\|}_{2}^{2} \end{array}\right\} \end{array}$$

Here, notation is conventionally abused such that the $L_{0}$ ‘pseudo-norm’ counts the number of nonzero elements in β .^10^

(2) $$\begin{array}{c} \|\beta {\|}_{0}:=\overset{p}{\underset{i=1}{\sum }}I\left\{\beta_{i}\neq 0\right\} \end{array}$$

Ridge regression^17^ penalises the model by the $L_{2}$ norm of the coefficients ( $\lambda_{0}=0,\lambda_{1}=0,\lambda_{2}\neq 0)$ , balancing predictive error against coefficient magnitude. The imposed preference for smaller coefficients is termed ‘shrinkage’. The magnitude of the shrinkage effect is controlled by the $\lambda_{2}$ hyperparameter.

(3) $${\hat{\beta }}^{Ridge}:=arg\underset{\left(\beta_{0},\beta \right)\in {\mathbb{R}}^{p+1}}{min}\left\{\frac{1}{2}{\left\|y-\beta_{0}-X\beta \right\|}_{2}^{2}+\lambda_{2}{\left\|\beta \right\|}_{2}^{2}\right\}$$

Ridge regression partially alleviates instability under collinearity by constraining coefficient magnitude.^16^ The Least Absolute Selection and Shrinkage Operator (LASSO)^11^ penalty penalises the model by the $L_{1}$ norm of the coefficients, ( $\lambda_{0}=0,\lambda_{1}\neq 0,\lambda_{2}=0)$ .

(4) $${\hat{\beta }}^{LASSO}:=arg\underset{\left(\beta_{0},\beta \right)\in {\mathbb{R}}^{p+1}}{min}\left\{\frac{1}{2}{\left\|y-\beta_{0}-X\beta \right\|}_{2}^{2}+\lambda_{1}{\left\|\beta \right\|}_{1}\right\}$$

The LASSO approach has ‘oracle’ properties under some conditions, meaning that predictions are nearly as good as if the true set of predictor variables were known.^18,19^ An additional benefit of LASSO shrinkage is a tendency to shrink small coefficients to zero, leading to a ‘sparse’ $\hat{\beta }$ , in which non-zero coefficients are deemed predictive. Thus, LASSO inherently performs variable selection.^11^ This behaviour is highly useful in bioinformatics, where analytic tasks often require the selection of a small number of predictive variables given a large candidate set. However, the lasso model structure is subject to inconsistency under subsampling.^12^ The Elastic Net^20^ is a combines the sparsity of $L_{1}$ penalisation with the consistency of $L_{2}$ penalisation ( $\lambda_{0}=0,\lambda_{1}\neq 0,\lambda_{2}\neq 0$ ), with improved results in several bioinformatic studies.^1,21^ Penalties of ridge regression, LASSO and elastic net affect large coefficients more than small coefficients, biassing coefficient estimates. ‘Best subset selection’ provides a theoretical solution to this issue through the selection of the optimal model attainable with k∈ℕ or fewer predictor variables, such that^10^:

(5) $$\begin{array}{c} {\hat{\beta }}^{BestSubset}:=arg\underset{\left(\beta_{0},\beta \right)\in {\mathbb{R}}^{p+1}}{min}\left\{\frac{1}{2}{\left\|y-\beta_{0}-X\beta \right\|}_{2}^{2}\right\}\\ subject\;to\left(\overset{p}{\underset{i=1}{\sum }}I\left\{\beta_{i}\neq 0\right\}\right)\leq k \end{array}$$

Thus, for some $\lambda_{0}\in \mathbb{R}$ , we have an equivalent Lagrangian expression:

(6) $$\begin{array}{c} {\hat{\beta }}^{BestSubset}:=arg\underset{\left(\beta_{0},\beta \right)\in {\mathbb{R}}^{p+1}}{min}\left\{\frac{1}{2}{\left\|y-\beta_{0}-X\beta \right\|}_{2}^{2}+\lambda_{0}{\left\|\beta \right\|}_{0}\right\} \end{array}$$

Best subset selection be may be approximated through $L_{0}$ penalisation in some conditions $\left(\lambda_{0}\neq 0,\lambda_{1}=0,\lambda_{2}=0\right)$ .^10^ $L_{0}$ penalisation applies no shrinkage to the selected predictors, resulting in unbiased coefficient estimates.^10^ This combination of simplicity and unbiasedness has been described as a ‘holy grail’ of sparse modelling.^9^ However, models suffer from inconsistency.^22^ Furthermore, issues such as non-convexity and NP-hardness complicate best-subset model selection.^9,23^ Recent developments such as mixed integer optimisation^10^ have facilitated best subset model learning. Combinations of $L_{0}$ penalties with $L_{1}$ ( $\lambda_{0}\neq 0,\lambda_{1}\neq 0,\lambda_{2}=0$ ) or $L_{2}$ ( $\lambda_{0}\neq 0,\lambda_{1}=0,\lambda_{2}\neq 0$ ) have been suggested to increase the consistency of best subset selection whilst maintaining minimal bias.^24^

### Assessing variable selection in genomic models

The true generating distribution for observational biological data is typically uncertain, complicating validation of estimated coefficient vectors. Consequently, many model assessments have employed synthetic^9,15,24-27^ or semi-synthetic^1,10,28-30^ datasets to assess variable selection performance. Real data analyses have focussed primarily on the models’ predictive capacity.^31-33^ Accurate predictions may not guarantee correct model structure, especially in the highly collinear conditions commonly encountered in genomics. The representativeness of synthe-tic datasets is both uncertain and untestable.^29^ Further-more, results of these studies have been discordant, suggesting dependence on the benchmark datasets and validation techniques.^9,10^

Genomic databases such as REACTOME^34^ and KEGG^35^ contain experimentally verified interactions, which may be used to externally validate genomic model structure. This approach has been used in previous analyses^27,29,36,37^ and is limited by the uncertain completeness of such databases. Furthermore, the activity profile of interactions between a given set of genes may change with experimental conditions and unobserved confounders.^38,39^ Consequently, the set of active predictors for a specific dataset may not align exactly with a static database. Finally, effect sizes may not be comparable between documented interactions, precluding the assessment of model coefficients by this method. Data-partitioning facilitates model validation without ground truth data, by assessing model generalisability to unseen observations. As training and validation observations are sampled from the same data, their distribution is asymptotically identical. However, the distribution may be difficult to estimate when n≪p , and data-partitioning favours excessively complex models in this setting.^14,15^

Given the limitations of currently available methods for assessment of variable selection performance in genomic data, an urgent need exists for a novel approach.

### Study objectives

The primary objectives of this study were to:

- Provide a real-world data-driven approach for comparing performance of high dimensional model inference algorithms in cancer genomics for both prediction and variable selection. We evaluate models by simulating n≪p conditions in real n>p genomic datasets, allowing for robust evaluation of predictions in large-sample test partitions.
- Compare the performance of penalised linear regression methods for prediction and variable selection.
- Compare algorithmic selection by internal cross-validation to preselection according to performance in external test datasets under our validation approach.

These objectives are realised by subsampling real n>p genomic datasets to simulate n≪p conditions, allowing for robust data-driven validation of model structure and predictions in large-sample test partitions.

## Materials and Methods

### Data

Five cancer genomics datasets were extracted from Gene Expression Omnibus^40^ with the GEOquery library.^41^ Local institutional review board approval and informed participant consent were documented in each data publication.^42-46^

### GSE73002

GSE73002^42^ contains serum miRNA expression profiles for 4113 individuals; 1280 with breast cancer, 54 with benign breast disease, 63 with non-benign breast disease, 451 with various other cancers and 2836 non-cancer controls. Participants with breast cancer were recruited through admissions and referrals to the National Cancer Centre Hospital Japan between 2008 and 2014. Exclusion criteria were (1) administration of medication prior to serum sampling and (2) advanced cancer in other organs. Controls were recruited from (1) National Cancer Centre Biobank, Yokohama Minoru clinic and the Toray Industries staff. Samples from individuals with non-benign breast diseases and other cancers were extracted from the National Cancer Centre Biobank. miRNA expression was measured with was collected on the Toray Industries 3D-Gene Human miRNA Oligo Chip microarray.

### GSE137140

GSE137140^43^ contains serum miRNA expression profiles for lung cancer patients. About 1566 pre-operative and 180 post-operative samples are available, in addition to 2178 samples from patients without cancer, collected from the National Cancer Centre Japan and the Yokohama Minoru Clinic. Exclusion criteria were (1) miRNA expression quality check failure, (2) history of other malignancy, (3) missing clinical information, (4) pre-collection therapy and (5) over 180 days had passed between collection and surgery. miRNA expression was measured with was collected on the Toray Industries 3D-Gene Human miRNA Oligo Chip microarray.

### GSE103322

GSE103322^44^ contains full length single-cell RNAseq data from 5902 cells extracted from 18 patients with stage I to IV squamous cell carcinoma (SCC) of the oral cavity at the Massachusetts Eye and Ear Infirmary. Tissue samples were extracted from surgical biopsies of the primary tumour or lymph node. Sequencing was performed on the Illumina Nextseq 500 platform and transcript-per-million values reported.

### GSE146026

GSE146026^45^ contains single-cell RNAseq data from 22 ascites samples in 11 patients with high-grade serous ovarian cancer at Brigham and Women’s Hospital and the Dana-Farber Cancer Institute. About 9609 CD45+ depleted samples, profiled with 10× were included in this analysis. Sequencing was performed on the Illumina NextSeq 500 platform and transcript-per-million values reported.

### GSE89567

GSE89567^46^ contains 6341 single-cell RNAseq profiles from patients with isocitrate dehydrogenase mutant astrocytoma at Massachusetts General Hospital. Tumour tissue was collected from surgical resections and malignancy confirmed under frozen section. Following disaggregation, profiling was performed by Smart-seq2. Sequencing was performed on the Illumina NextSeq 500 and transcript-per-million values reported.

### Data preprocessing

Where datasets had >5000 variables (GSE103322 and GSE146026), subspaces were extracted, retaining the 1000 variables with the fewest nonzero entries. Datasets were transformed with the Gaussian ECDF function^47,48^:

(7) $$\begin{array}{c} X_{i,j}:=\Phi^{-1}\left(\frac{1}{n}\overset{n}{\underset{k=1}{\sum }}I\left\{X_{k,j}\leq X_{i,j}\right\}\right) \end{array}$$

Here Φ(⋅) is the standard normal cumulative distribution function. To ensure uniqueness of the gold-standard model, QR-factorisation was performed, and perfectly collinear variables were removed.

(8) $$\begin{array}{c} X=QRP^{T} \end{array}$$

Here Q is an orthogonal matrix, R is an upper triangular matrix and P is a permutation matrix. A full-rank subspace was extracted from X using QR factorisation, such that:

(9) $$\begin{array}{c} X:=XP_{:,i\leq rank\left(X\right)}^{T} \end{array}$$

### Experiment setup

In each experiment, 500 design variables and a response were randomly selected from the available gene expression variables in 1 of the 5 datasets. A small proportion of the observations (n∈{25,75,150}) were randomly selected for training and the remainder held out for validation. $L_{0},L_{0}L_{1},L_{0}L_{2},L_{1}$ and $L_{1}L_{2}$ penalised regression models were fitted using default library parameters (Table 1). Regularisation hyperparameters were selected by either 5-fold or 10-fold cross-validation on the training observations, optimising the mean squared error, a typical approach in genomic analyses.^1,6,7,49,50^ The same cross-validation folds were employed for each penalisation method in a given experiment. Predictive performance and variable selection performance were assessed using the remaining test observations. Experiments were repeated for 100 different training samples, for each of 5 datasets and for both cross-validation routines, yielding 1000 experiments with which to compare penalisation methods for each sample size.

**Table 1.** Penalised regression methods applied in this analysis.

Pseudonym	Notation	Penalty	Implementation	Reference
Best-subset selection	*L*_0_	*λ*_0_ ≠ 0, *λ*_1_ = 0, *λ*_2_ = 0	*L*_0_Learn 1.2.0^24^	Hastie et al^9^ and Bertsimas et al^10^
			Loss = ‘SquaredError’	
			Penalty = ‘L0’	
			Algorithm = ‘CD’	
			Nlambda = 100	
			nGamma = 10	
			gammaMax = 10	
			gammaMin = 1e-04	
			partialSort = TRUE	
			maxIters = 200	
			tol = 1e-06	
			activeset = TRUE	
			activesetnum = 3	
			maxswaps = 1000	
			scaledownFactor = 0.8	
			screenSize = 1000	
			autoLambda = TRUE	
			nFolds = 5	
			excludeFirstK = 0	
			intercept = FALSE	
*L*_0_ *L*_1_	$L_{0}L_{1}$	$\lambda_{0}\neq 0,\lambda_{1}\neq 0,\lambda_{2}=0$	*L*_0_Learn 1.2.0	Hazimeh and Mazumder^24^
			Same as above except: Penalty = ‘*L*_0_*L*_1_’	
*L*_0_ *L*_1_	$L_{0}L_{2}$	$\lambda_{0}\neq 0,\lambda_{1}=0,\lambda_{2}\neq 0$	*L*_0_Learn 1.2.0	Hazimeh and Mazumder^24^
			Same as above except: Penalty = ‘*L*_0_*L*_2_’	
LASSO	$L_{1}$	$\lambda_{0}=0,\lambda_{1}\neq 0,\lambda_{2}=0$	glmnet 4.2-0^51,52^	Tibshirani^11^
			family = ‘gaussian’	
			alpha = 1	
			weights = NULL	
			offset = NULL	
			lambda = NULL	
			lambda.min.ratio = 1e-4	
			type.measure = ‘mse’	
			foldid = NULL	
			alignment = ‘lambda’	
			grouped = TRUE	
			relax = FALSE	
			alpha = 0	
			parallel = FALSE	
Elastic net	*L*_1_ *L*_2_	$\lambda_{0}=0,\lambda_{1}\neq 0,\lambda_{2}\neq 0$	glmnet 4.2-0	Zou and Hastie^20^
			Same as above except: alpha = {0, 0.11, 0.22, 0.33, 0.44, 0.56, 0.67, 0.78, 0.89, 1}	

λ Notation corresponds to the regularisation hyperparameters defined in equation (1).

### Metrics

Model assessment metrics and notation followed previous comparative analyses.^9,10^ As the true coefficient vector, $\beta \in {\mathbb{R}}^{p}$ , was unknown in our experiments, it was estimated by ordinary least squares regression (without intercept) on the whole dataset (n≈4000,p=500) , such that:

(10) $$\begin{array}{c} \beta \approx \beta^{*}={\left(X^{T}X\right)}^{-1}X^{T}y \end{array}$$

Thus, $\beta^{*}$ represents a noisy gold-standard rather than strict ground truth. Here $x_{0}\in {\mathbb{R}}^{p}$ denotes the test observations from the design matrix and $y_{0}\in \mathbb{R}$ denotes the associated response. Hastie et al^9^ measured 3 metrics of predictive performance – proportion of variance explained (PVE), relative risk (RR) and relative test error (RTE).

(11) $$\begin{array}{c} PVE\left(\hat{\beta }\right)=1-\frac{E\left[{\left(y_{0}-x_{0}^{T}\hat{\beta }\right)}^{2}\right]}{Var\left(y_{0}\right)} \end{array}$$

Higher PVE indicates superior fit, and PVE is limited by the signal to noise ratio (SNR) such that^9^:

(12) $$\begin{array}{c} PVE\left(\hat{\beta }\right)\leq \frac{SNR}{1+SNR}\leq 1 \end{array}$$

Relative risk (RR) was employed as an performance metric in Bertsimas’ analysis.^10^ Optimal relative risk is 0 and nullity is 1.

(13) $$\begin{array}{c} RR\left(\hat{\beta }\right)=\frac{E\left[{\left(x_{0}^{T}\beta -x_{0}^{T}\hat{\beta }\right)}^{2}\right]}{E\left[{\left(x_{0}^{T}\beta^{*}\right)}^{2}\right]} \end{array}$$

Relative test error (RTE) compares error to the noise variance:

(14) $$\begin{array}{c} RTE\left(\hat{\beta }\right)=\frac{E\left[{\left(y_{0}-x_{0}^{T}\hat{\beta }\right)}^{2}\right]}{E\left[{\left(y_{0}-x_{0}^{T}\beta^{*}\right)}^{2}\right]} \end{array}$$

Following calls for model coefficient similarity assessment,^9^ we measured the cosine similarity of $\hat{\beta }$ and $\beta^{*}$ , such that:

(15) $$\begin{array}{c} CoefficientSimilarity\left(\hat{\beta }\right)=\frac{〈\hat{\beta },\beta^{*}〉}{\sqrt{〈\hat{\beta },\hat{\beta }〉〈\beta^{*},\beta^{*}〉}} \end{array}$$

Active (non-zero) variable selection performance was also estimated under $\beta^{*}.$ Coefficient significance of was estimated with *t*-tests:

(16) $$\begin{array}{c} \mathbb{P}\left(\beta_{i}^{*}=0\right)\sim t_{n-p}\left(\beta_{i}^{*}\right)=\frac{\beta_{i}^{*}}{SE\left(\beta_{i}^{*}\right)} \end{array}$$

**Figure 1.**
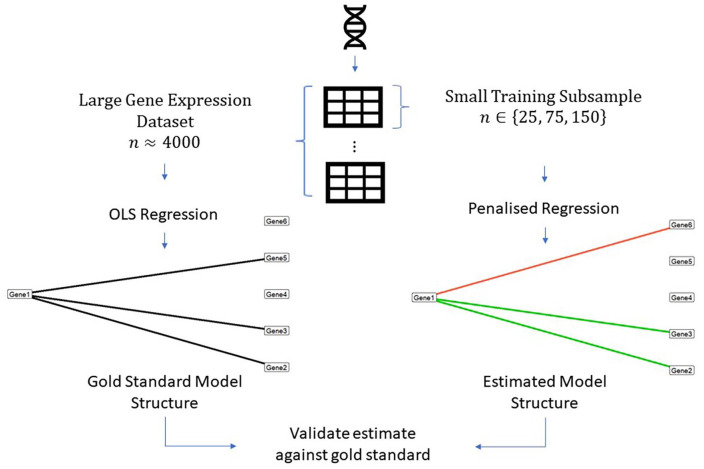
Graphical visualisation of variable selection validation method. ‘Gold-standard’ regression models were trained on subspaces of large genomic datasets ( n4000 , p=500 ). *T*-tests were performed on gold standard coefficient estimates and significant coefficients were identified according to a false-discovery rate controlled alpha cutoff of .05. Penalised regression models were trained on small samples from these subspaces ( n∈{25,75,150},p=500 ) and validated against the gold-standard models.

Significance was adjusted for multiple comparisons using false-discovery-rate (FDR) control^53^ and predictors were classified according to a cutoff α=0.05 . Precision, recall, F1 score were measured. Hereafter, these metrics are referred to collectively as the ‘discrete’ variable selection metrics. Undefined variable selection results (due to division-by-zero errors) were replaced with zeros. Figure 1 depicts the variable selection validation method graphically.

To evaluate our model validation approach, we deployed it as a penalty preselection method, comparing it to traditional selection by minimisation of the internal cross-validation error. For each experiment, for each of 3 comparison metrics (PVE, F1 and coefficient similarity), a penalisation method was ‘preselected’ according to performance in experiments of equivalent sample size in the other 4 datasets. In each relevant experiment, penalisation methods’ performances were ranked and the method with the lowest rank aggregate performance was selected. The test performance of this method was compared to that of the penalisation method which yielded the lowest mean squared error on internal cross-validation. Overall performance of preselected penalties was compared to internal cross-validation selected penalties using a 2-sided paired *t*-test over all 3000 experiments.

## Results

### Experiment characteristics

Experiments represented a broad range of signal:noise ratios (Median: 0.94, IQR: [0.38, 2.68]), with high SNR in experiments sampled from GSE73002 (Median: 12.03, IQR: [4.69, 28.34]), intermediate SNR in GSE137140 (Median: 1.58, IQR: [1.16, 2.41]) and low SNRs in GSE103322 (Median: 0.47, IQR: [0.31, 0.85]), GSE146026 (Median: 0.39, IQR: [0.23, 0.77]) and GSE89567 (Median: 0.44, IQR: [0.31, 0.71]). The number of significant coefficients in each experiment was typically small (Median: 7.00, IQR: [1.00, 17.00]) and followed a right-skewed distribution (95th Quantile: 40.00, Max: 106.00). This is consistent with the scale-free property of genomic networks, in which a small number of genes have many interactions.

### Predictive performance

**Figure 2.**
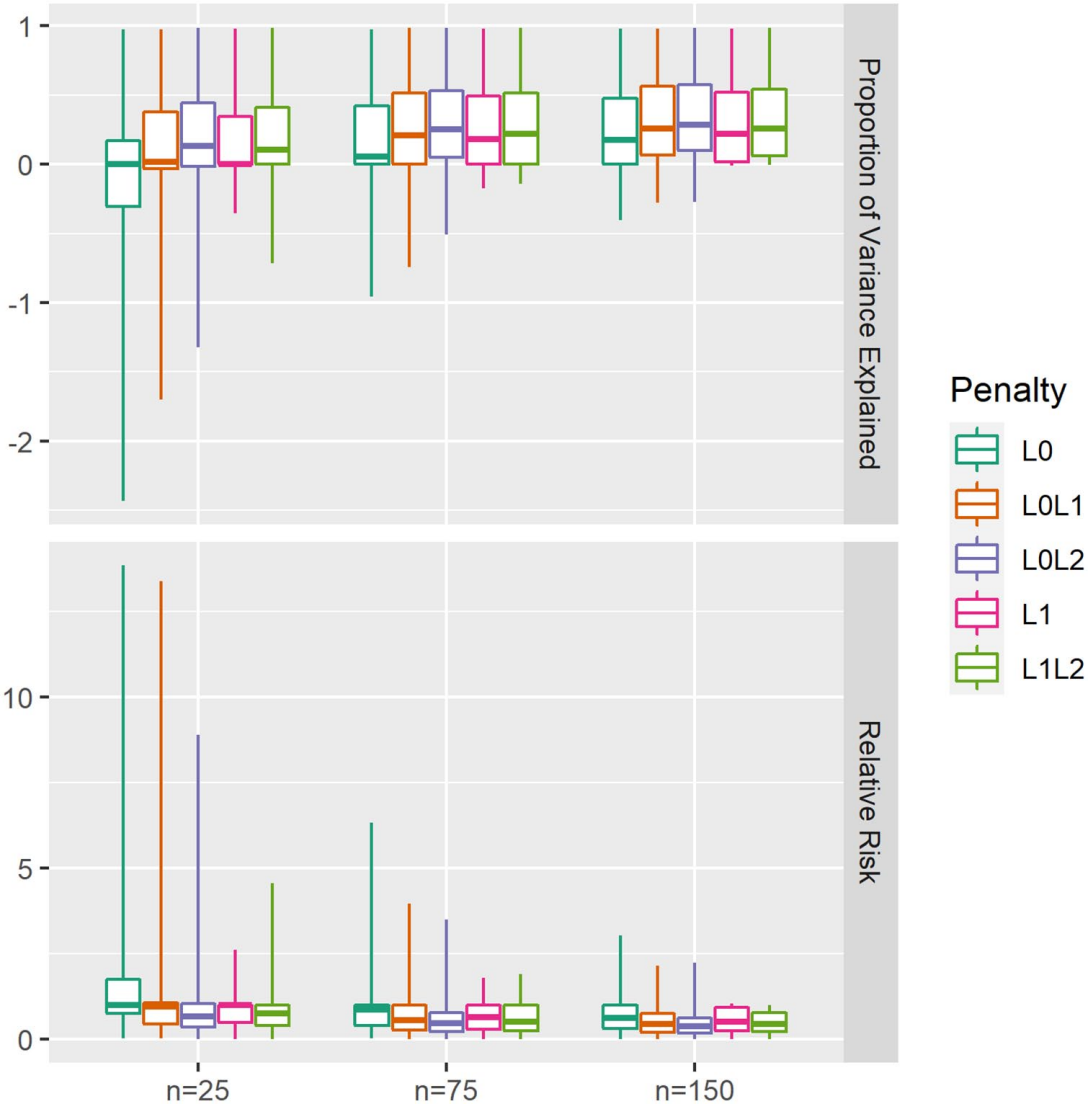
Test predictive performance. Medians are represented by boxplot centrelines; first and third quartiles by hinges; and minima and maxima by whiskers.

**Figure 3.**
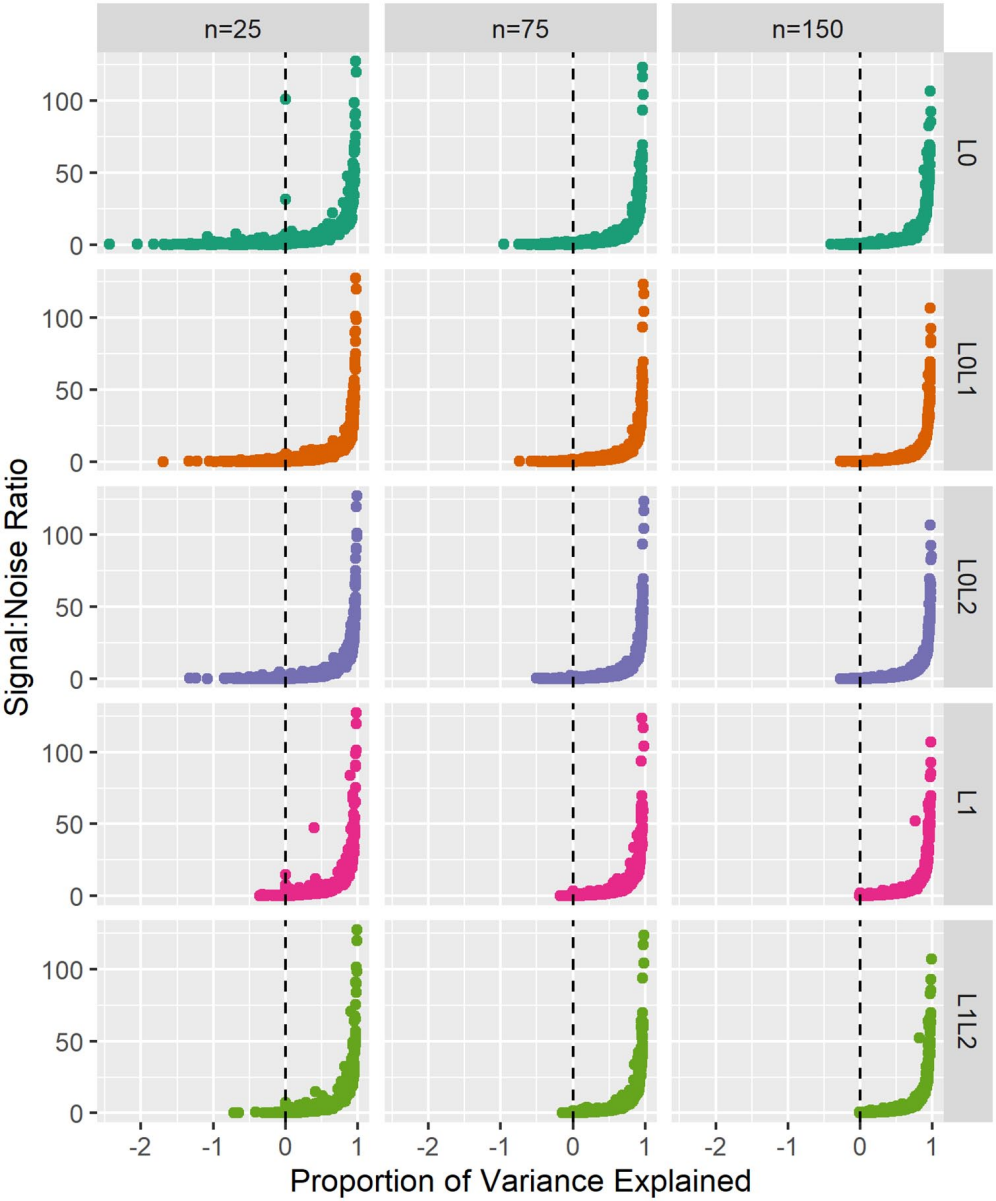
Proportion of variance explained in test observations versus signal:noise ratio. Signal:noise ratio was estimated by the residuals of the gold standard models fitted to the complete dataset (n4000 ) with ordinary least squares regression. Medians are represented by boxplot centrelines; first and third quartiles by hinges; and minima and maxima by whiskers.

Predictive performance metrics are provided in Figure 2 and Table 2. $L_{0}L_{2}$ -penalised models achieved the highest PVE overall (Median: 0.23, IQR: [0.04, 0.52]). However, this penalty performed unreliably in the n = 25 experiments, demonstrating strongly negative PVE values (ie, worse-than-random performance) in some cases (Min: −1.32, 5th Quantile: −0.33). Similarly, $L_{0}L_{1}$ -penalised models exhibited strong overall PVE (Median: 0.17, IQR: [−0.00, 0.50]) and variable performance in the n = 25 setting (Min: −1.70, 5th Quantile: −0.39). L1L2 penalised models achieved comparable overall PVE (Median: 0.19, IQR: [−0.00, 0.49]), with superior worst-case reliability in the n = 25 experiments (Min: −0.71, 5th Quantile: −0.01). Likewise, $L_{1}$ penalisation provided moderate overall PVE (Median: 0.13, IQR: [−0.00, 0.47]) and robust worst-case PVE scores in the n = 25 experiments (Min: −0.35, 5th Quantile: −0.01). $L_{0}$ penalisation selected null models in most experiments, returning null PVE (Median: 0.01, IQR: [−0.01, 0.40]). PVE was highly associated with SNR (: 0.61, 95% CI: [0.6, 0.62], *P* < 2e-16. PVE:SNR curves (Figure 3) demonstrate that $L_{0}L_{1}$ and $L_{0}L_{2}$ underperformance was mainly limited to the noisiest cases. $L_{1}$ and $L_{1}L_{2}$ penalisation were infrequently negative, even in noisy experiments. Conversely, $L_{1}$ and $L_{1}L_{2}$ penalisation demonstrated poorer PVE reliability than $L_{0}L_{1}$ and $L_{0}L_{2}$ penalisation in moderate SNR conditions. Relative risk performance distributions reflected those of PVE, with the best overall median performance observed in $L_{0}L_{2}$ (Median: 0.48, IQR: [0.24, 0.81]) and $L_{0}L_{1}$ -penalised models (Median: 0.58, IQR: [0.28, 1.00]), despite unreliable worst-case performance observed in n = 25 settings. Moderate relative risk performance was achieved through $L_{1}$ (Median: 0.68, IQR: [0.31, 1.00]) and $L_{1}L_{2}$ penalisation (Median: 0.23, IQR: [0.04, 0.52]), with superior worst-case reliability. RTE performance highlighted the shortcomings of *L*0 penalisation (Median: 1.79, IQR: [1.45, 2.33]).

**Table 2.** Predictive performance of each penalisation method.

Penalty	N	Metric	Median	IQR
*L*_0_	25	Proportion of variance explained	0	[0.00, 0.00]
*L*_0_ *L*_1_	25	Proportion of variance explained	0	[0.00, 0.06]
*L*_0_ *L*_2_	25	Proportion of variance explained	0	[0.00, 0.08]
*L*_1_	25	Proportion of variance explained	0	[0.00, 0.00]
*L*_1_ *L*_2_	25	Proportion of variance explained	0	[0.00, 0.06]
*L*_0_	75	Proportion of variance explained	0	[0.00, 0.20]
*L*_0_ *L*_1_	75	Proportion of variance explained	0	[0.00, 0.16]
*L*_0_ *L*_2_	75	Proportion of variance explained	0.02	[0.00, 0.14]
*L*_1_	75	Proportion of variance explained	0	[0.00, 0.22]
*L*_1_ *L*_2_	75	Proportion of variance explained	0.02	[0.00, 0.09]
*L*_0_	150	Proportion of variance explained	0	[0.00, 0.33]
*L*_0_ *L*_1_	150	Proportion of variance explained	0.05	[0.00, 0.20]
*L*_0_ *L*_2_	150	Proportion of variance explained	0.05	[0.00, 0.18]
*L*_1_	150	Proportion of variance explained	0.07	[0.00, 0.29]
*L*_1_ *L*_2_	150	Proportion of variance explained	0.02	[0.00, 0.10]
*L*_0_	25	Relative risk	1	[0.75, 1.75]
*L*_0_ *L*_1_	25	Relative risk	0.94	[0.45, 1.07]
*L*_0_ *L*_2_	25	Relative risk	0.67	[0.36, 1.04]
*L*_1_	25	Relative risk	1	[0.48, 1.00]
*L*_1_ *L*_2_	25	Relative risk	0.75	[0.39, 1.00]
*L*_0_	75	Relative risk	0.87	[0.40, 1.00]
*L*_0_ *L*_1_	75	Relative risk	0.56	[0.26, 1.00]
*L*_0_ *L*_2_	75	Relative risk	0.46	[0.23, 0.78]
*L*_1_	75	Relative risk	0.63	[0.29, 1.00]
*L*_1_ *L*_2_	75	Relative risk	0.52	[0.26, 1.00]
*L*_0_	150	Relative risk	0.62	[0.31, 1.00]
*L*_0_ *L*_1_	150	Relative risk	0.44	[0.20, 0.75]
*L*_0_ *L*_2_	150	Relative risk	0.37	[0.19, 0.61]
*L*_1_	150	Relative risk	0.52	[0.24, 0.93]
*L*_1_ *L*_2_	150	Relative risk	0.44	[0.22, 0.77]
*L*_0_	25	Relative test error	0	[−0.30, 0.17]
*L*_0_ *L*_1_	25	Relative test error	0.02	[−0.03, 0.38]
*L*_0_ *L*_2_	25	Relative test error	0.13	[−0.02, 0.44]
*L*_1_	25	Relative test error	0	[−0.00, 0.34]
*L*_1_ *L*_2_	25	Relative test error	0.1	[−0.00, 0.41]
*L*_0_	75	Relative test error	0.06	[−0.00, 0.42]
*L*_0_ *L*_1_	75	Relative test error	0.21	[−0.00, 0.52]
*L*_0_ *L*_2_	75	Relative test error	0.25	[0.05, 0.53]
*L*_1_	75	Relative test error	0.18	[−0.00, 0.49]
*L*_1_ *L*_2_	75	Relative test error	0.22	[−0.00, 0.51]
*L*_0_	150	Relative test error	0.18	[−0.00, 0.48]
*L*_0_ *L*_1_	150	Relative test error	0.26	[0.07, 0.56]
*L*_0_ *L*_2_	150	Relative test error	0.28	[0.10, 0.57]
*L*_1_	150	Relative test error	0.22	[0.02, 0.52]
*L*_1_ *L*_2_	150	Relative test error	0.26	[0.06, 0.54]

Abbreviation: IQR, interquartile range.

For each sample size, 100 experiments were sampled from each of 5 datasets, for each of 2 cross-validation routines. IQR denotes interquartile range.

### Variable selection

**Figure 4.**
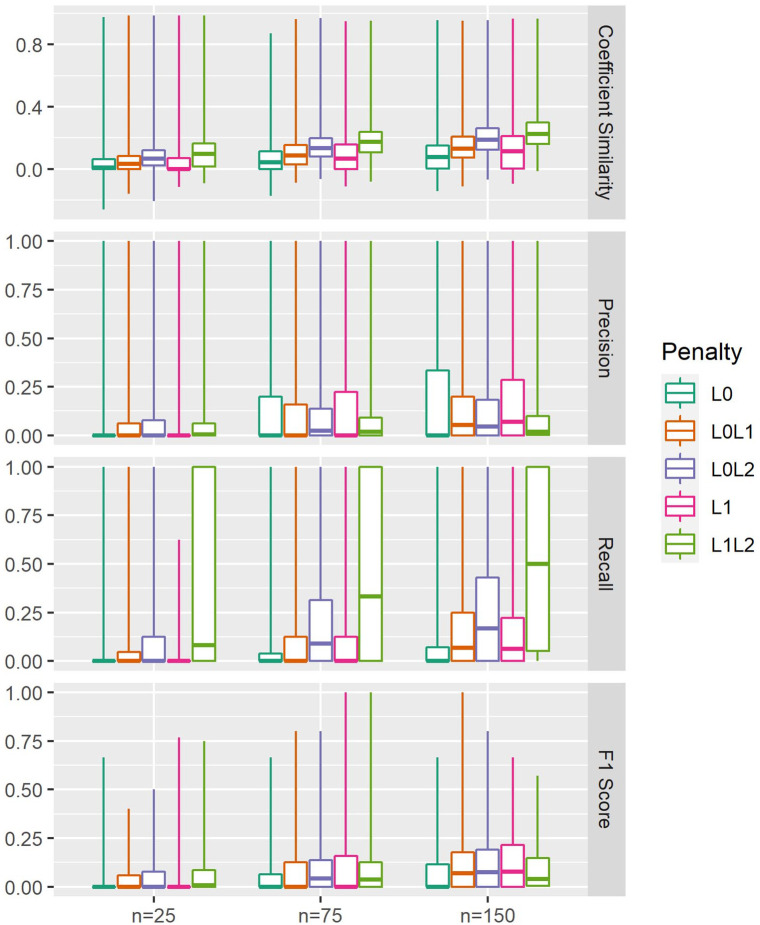
Variable selection performance. Gold standard coefficient vectors were extracted from ordinary least squares regression models fitted to the full dataset (n4000) . Coefficient significance was estimated with *t*-tests and true predictors were defined by FDR- α<0.05 . Medians are represented by boxplot centrelines; first and third quartiles by hinges; and minima and maxima by whiskers.

Variable selection performance metrics are provided in Figure 4 and Table 3. $L_{1}L_{2}$ -penalised models achieved high coefficient similarity overall (Median: 0.17, IQR: [0.09, 0.24]), although many nonzero coefficients were included (Median: 59.50, IQR: [11.00, 500.00]). Consequently, in n = 75 experiments, strong recall (Median: 0.33, IQR: [0.00, 1.00]) and poor precision were observed (Median: 0.33, IQR: [0.00, 1.00]). $L_{0}L_{2}$ -penalisation also achieved high coefficient similarity (Median: 0.13, IQR: [0.06, 0.20]), with fewer nonzero coefficients (Median: 25.00, IQR: [6.00, 67.00]). $L_{0}L_{2}$ penalisation achieved the highest F1 score in n = 75 (Median: 0.04, IQR: [0.00, 0.14]) and n = 150 experiments (Median: 0.07, IQR: [0.00, 0.19]). $L_{0}L_{1}$ -penalised models performed similarly in terms of coefficient similarity (Median: 0.08, IQR: [0.02, 0.15]) using fewer nonzero parameters (Median: 8.00, IQR: [2.00, 19.00]). Moderate F1 scores were achieved in n = 75 and (Median: 0.00, IQR: [0.00, 0.12]) and n = 150 experiments (Median: 0.07, IQR: [0.00, 0.18]) $L_{1}$ -penalised models achieved moderate coefficient similarity (Median: 0.05, IQR: [0.00, 0.15]) through models with very few nonzero coefficients (Median: 5.00, IQR: [0.00, 10.00]). Although $L_{1}$ -penalisation achieved moderate F1 score in n = 150 experiments (Median: 0.08, IQR: [0.00, 0.21]), it underperformed in n = 75 experiments (Median: 0.00, IQR: [0.00, 0.16]). $L_{0}$ -only penalisation produced highly parsimonious models, with very few nonzero coefficients (Max: 67.00, 95th Quantile: 10.05). However, variable selection performance was poor by every metric. Test performance summaries for prediction and variable selection are provided in Supplemental Table S1.

**Table 3.** Variable selection performance of each penalisation method.

Penalty	N	Metric	Median	IQR
*L*_0_	25	Coefficient similarity	2	[0.00, 3.00]
*L*_0_ *L*_1_	25	Coefficient similarity	6	[1.00, 16.00]
*L*_0_ *L*_2_	25	Coefficient similarity	16	[4.00, 42.00]
*L*_1_	25	Coefficient similarity	1	[0.00, 6.00]
*L*_1_ *L*_2_	25	Coefficient similarity	25.5	[4.00, 500.00]
*L*_0_	75	Coefficient similarity	2	[0.00, 3.00]
*L*_0_ *L*_1_	75	Coefficient similarity	8	[2.00, 20.00]
*L*_0_ *L*_2_	75	Coefficient similarity	27.5	[6.00, 75.00]
*L*_1_	75	Coefficient similarity	5	[0.00, 10.00]
*L*_1_ *L*_2_	75	Coefficient similarity	98.5	[13.00, 500.00]
*L*_0_	150	Coefficient similarity	3	[1.00, 4.00]
*L*_0_ *L*_1_	150	Coefficient similarity	12	[3.00, 23.00]
*L*_0_ *L*_2_	150	Coefficient similarity	35	[10.00, 79.00]
*L*_1_	150	Coefficient similarity	8	[1.00, 13.00]
*L*_1_ *L*_2_	150	Coefficient similarity	500	[20.00, 500.00]
*L*_0_	25	F1 score	0.01	[0.00, 0.06]
*L*_0_ *L*_1_	25	F1 score	0.03	[0.00, 0.08]
*L*_0_ *L*_2_	25	F1 score	0.07	[0.02, 0.12]
*L*_1_	25	F1 score	0	[0.00, 0.07]
*L*_1_ *L*_2_	25	F1 score	0.1	[0.02, 0.16]
*L*_0_	75	F1 score	0.04	[0.00, 0.11]
*L*_0_ *L*_1_	75	F1 score	0.08	[0.03, 0.15]
*L*_0_ *L*_2_	75	F1 score	0.13	[0.08, 0.20]
*L*_1_	75	F1 score	0.07	[0.00, 0.16]
*L*_1_ *L*_2_	75	F1 score	0.17	[0.11, 0.24]
*L*_0_	150	F1 score	0.07	[0.00, 0.15]
*L*_0_ *L*_1_	150	F1 score	0.13	[0.07, 0.21]
*L*_0_ *L*_2_	150	F1 score	0.19	[0.12, 0.26]
*L*_1_	150	F1 score	0.11	[0.00, 0.21]
*L*_1_ *L*_2_	150	F1 score	0.22	[0.16, 0.30]
*L*_0_	25	Precision	0	[0.00, 0.00]
*L*_0_ *L*_1_	25	Precision	0	[0.00, 0.05]
*L*_0_ *L*_2_	25	Precision	0	[0.00, 0.12]
*L*_1_	25	Precision	0	[0.00, 0.00]
*L*_1_ *L*_2_	25	Precision	0.08	[0.00, 1.00]
*L*_0_	75	Precision	0	[0.00, 0.04]
*L*_0_ *L*_1_	75	Precision	0	[0.00, 0.12]
*L*_0_ *L*_2_	75	Precision	0.09	[0.00, 0.31]
*L*_1_	75	Precision	0	[0.00, 0.12]
*L*_1_ *L*_2_	75	Precision	0.33	[0.00, 1.00]
*L*_0_	150	Precision	0	[0.00, 0.07]
*L*_0_ *L*_1_	150	Precision	0.07	[0.00, 0.25]
*L*_0_ *L*_2_	150	Precision	0.17	[0.00, 0.43]
*L*_1_	150	Precision	0.06	[0.00, 0.22]
*L*_1_ *L*_2_	150	Precision	0.5	[0.05, 1.00]
*L*_0_	25	Recall	0	[0.00, 0.00]
*L*_0_ *L*_1_	25	Recall	0	[0.00, 0.06]
*L*_0_ *L*_2_	25	Recall	0	[0.00, 0.08]
*L*_1_	25	Recall	0	[0.00, 0.00]
*L*_1_ *L*_2_	25	Recall	0.01	[0.00, 0.09]
*L*_0_	75	Recall	0	[0.00, 0.06]
*L*_0_ *L*_1_	75	Recall	0	[0.00, 0.12]
*L*_0_ *L*_2_	75	Recall	0.04	[0.00, 0.14]
*L*_1_	75	Recall	0	[0.00, 0.16]
*L*_1_ *L*_2_	75	Recall	0.04	[0.00, 0.12]
*L*_0_	150	Recall	0	[0.00, 0.12]
*L*_0_ *L*_1_	150	Recall	0.07	[0.00, 0.18]
*L*_0_ *L*_2_	150	Recall	0.07	[0.00, 0.19]
*L*_1_	150	Recall	0.08	[0.00, 0.21]
*L*_1_ *L*_2_	150	Recall	0.04	[0.00, 0.15]

Abbreviation: IQR, interquartile range.

For each sample size, 100 experiments were sampled from each of 5 datasets, for each of 2 cross-validation routines, yielding 1000 experiments for each comparison.

### Comparing preselection to internal validation

**Figure 5.**
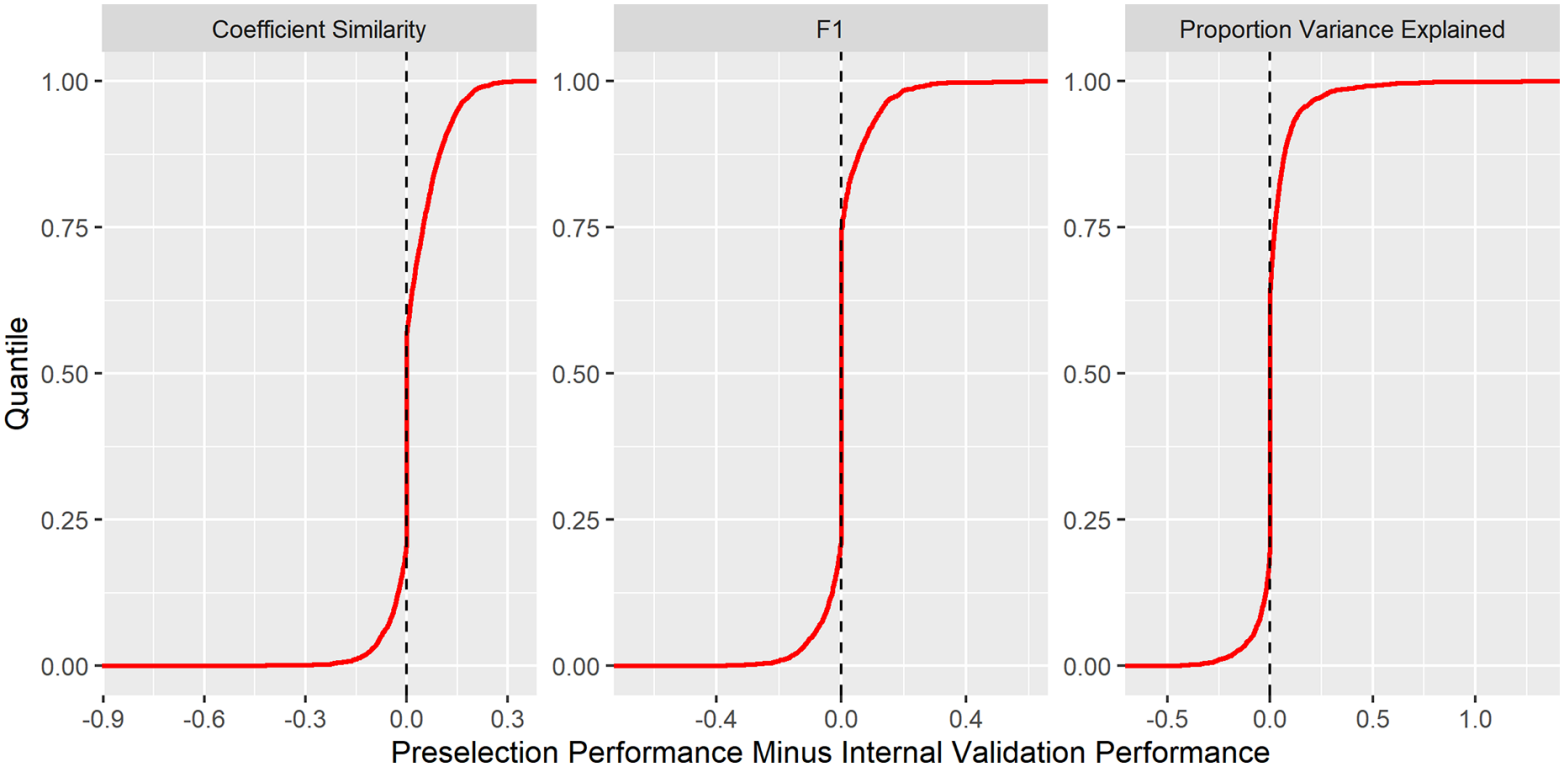
Cumulative distribution functions for performance improvement under penalty preselection compared with comparison to selection by internal cross-validation. For each experiment and each comparison metric, the penalisation method was selected with the best test performance in the other 4 datasets. This ‘preselected’ penalisation method was compared to that which minimised the mean squared error in internal cross-validation. About 3000 experiments were included in the comparison.

Penalty preselection led to small, yet significant, performance gains in PVE ( $t_{2999}:$ 8.66, µ: 0.016, 95% CI: [0.012, 0.020], P < 10^−16^), F1 score ( $t_{2999}:$ 4.66, µ: 0.016, 95% CI: 0.006 [0.003, 0.008], *P* = 3.3 × 10^−6^) and coefficient similarity ( $t_{2999}:$ 15.99, µ: 0.02, 95% CI: [0.018, 0.023], P < 10^−16^) when compared to selection by internal cross-validation. In many cases the same penalisation method was selected under preselection and internal validation, leading to equivalent performance. Although aggregated improvements under preselection were statistically significant, internal validation outperformed in some experiments (Figure 5). Cumulative distribution functions of the performance improvements yielded under preselection are provided in (Figure 5). In other experiments internal validation outperformed preselection (Table 4).

**Table 4.** Paired-tests of mean performance difference using preselection compared to selection by internal cross-validation. Penalisation routines were ‘preselected’ according to performance in the other 4 datasets. Mean difference refers to preselected performance minus internal cross-validation performance. About 3000 experiments were included in the comparison.

Metric	*t*-Score (*df* = 2999)	Mean performance gain under preselection	95% CI	*P*-value (2-sided)
Proportion of variance explained	8.66	0.016	[0.012, 0.020]	$<10^{-16}$
F1	4.66	0.006	[0.003, 0.008]	$3.3\times 10^{-6}$
Coefficient similarity	15.99	0.020	[0.018, 0.023]	$<10^{-16}$

## Discussion

The optimal penalisation method for a particular dataset depends upon the project objectives, data distribution and noise levels. In most applications, reliability is paramount – the strong median predictive performance provided by $L_{0}L_{1}$ and $L_{0}L_{2}$ penalisation is unlikely to compensate for their worst-case performance, which may be undetectable in application. $L_{1}L_{2}$ penalisation offered strong coefficient similarity, though few coefficients are shrunk to zero, limiting its utility for the selection of parsimonious model structures. $L_{1}$ and $L_{1}L_{2}$ penalties also offered reliable test predictions in noisy data. $L_{1}$ is simpler to implement than combined penalties, requiring tuning of a single hyperparameter. Furthermore, the theory surrounding $L_{1}$ penalisation in the n≪p setting is well studied.^1,7,12,54^ Various computational implementations of this method are available, and it is the fundamental building block for graph inference methods such as the graphical LASSO^55^ and the nodewise LASSO.^56^ $L_{0}$ penalisation resulted in weakly predictive models and poor variable selection, due primary to inadequate recall. These limitations overshadowed any potential advantage of theoretical unbiasedness.^10^

Penalty preselection yielded small, yet significant improvements over internal cross-validation based selection in each examined metric, demonstrating the value of external data-driven preselection of model learning algorithms for n≪p datasets. This approach may serve as a complementary methodological validation measure for genomic datasets.

### Related work

Bertsimas et al^10^ found that $L_{0}$ penalisation outperformed the $L_{1}$ and forward stepwise regression in their comparisons. However, this result was contested in the comparisons of Hastie et al,^9^ who concluded that $L_{1}$ outperformed $L_{0}$ in all but high signal-to-noise conditions. Hazimeh and Mazumder^24^ found that $L_{0}L_{1}$ and $L_{0}L_{2}$ penalties typically outperformed $L_{1}$ ,^24^ a finding which concurs with our experiments.

### Limitations

The primary limitation of this analysis is uncertainty regarding the true generating distributions of the datasets. In place of ground truth, a ‘gold-standard’ was set using a much larger number of observations. Thus, our analysis evaluates its capacity to recover the model which would have been found in a much larger study of the same population, a reasonable objective in many clinical studies. As the gold standard models were fitted to a finite number of observations, they were susceptible to some degree of overfitting.

Observations were not strictly partitioned on a patient-disjoint basis. In the typical clinical modelling scenario, estimation of model generalisability to new patients would require patient-disjoint partitioning and validation.^57^ However, distributional identicality of the training and test data would not have been guaranteed in such conditions, biassing assessment metrics in favour of underfitted models.

Bertsimas and Hastie both considered which SNR ranges were ‘realistic’; Bertsimas generated tasks with SNR ∈[2,10] and Hastie examined the SNR ∈[0.05,6] setting.^9,10^ Our estimated SNRs align with those of Hastie. In the case that the gold standard models overfitted, noise levels would have been underestimated. Therefore, SNR estimates in this analysis are positively biassed. Nonzero coefficients were defined according to a traditional, yet arbitrary significance cutoff – therefore small effects may have been omitted erroneously. Likewise, some spuriously large coefficients may have been included.

Discrete variable selection metrics (precision, recall and F1 score) lacked the graduation required to compare penalisation methods at the n = 25 level. This limitation was particularly important in the setting of active variables estimated according to a sharp significance cutoff. The coefficient similarity metric proved useful in this regard, as it was continuous and independent of any significance cutoff. However, coefficient similarity provides little insight into on model complexity, a central aspect of genomic network inference. Indeed, although $L_{1}L_{2}$ penalisation optimised the coefficient similarity metric, it selected extremely complex models in most experiments, resulting in weak precision.

Real-world genomic datasets were employed in this analysis. Accordingly, our results are expected to be more representative of actual experimental modelling conditions. Data-driven model assessment was facilitated by the large number of observations available in these datasets. However, our results may not generalise to datasets with incomparably distributed signal or noise. Logistic and Cox regression tasks present addition challenges such as class imbalance and censoring, which are beyond the scope of this analysis.

## Conclusions

$L_{0}L_{2}$ -penalised model provided the best test predictions, though performance was unreliable in noisy data. $L_{0}L_{2}$ also optimised discrete variable selection metrics. $L_{1}L_{2}$ -penalisation returned offered reliable test predictions in all settings and superior coefficient similarity. Further research is required to establish the performance of the penalties in classification and survival tasks. Evaluation of learning algorithms according to observed test performance in external genomic datasets yields valuable insights into actual test performance, providing a data-driven complement to internal cross-validation in genomic regression tasks.

## Supplemental Material

sj-txt-1-cix-10.1177_11769351211056298 – Supplemental material for Sparse Regression in Cancer Genomics: Comparing Variable Selection and Predictions in Real World Data Click here for additional data file. Supplemental material, sj-txt-1-cix-10.1177_11769351211056298 for Sparse Regression in Cancer Genomics: Comparing Variable Selection and Predictions in Real World Data by Robert J O’Shea, Sophia Tsoka, Gary JR Cook and Vicky Goh in Cancer Informatics
